# Clinical features of suspected Ebola cases referred to the Moyamba ETC, Sierra Leone: challenges in the later stages of the 2014 outbreak

**DOI:** 10.1186/s12879-016-1609-9

**Published:** 2016-06-22

**Authors:** Javier Arranz, Karen Marie Lundeby, Shoaib Hassan, Luis Matías Zabala Fuentes, Pedro San José Garcés, Yngvar Lunde Haaskjold, Håkon Angell Bolkan, Kurt Østhuus Krogh, James Jongopi, Sindre Mellesmo, Ola Jøsendal, Åsmund Øpstad, Erling Svensen, Alfred Sandy Kamara, David P. Roberts, Paul D. Stamper, Paula Austin, Alfredo J. Moosa, Dennis Marke, Åse Berg, Bjørn Blomberg, Melcior Riera

**Affiliations:** Médicos del Mundo, Madrid, Spain; Arquitecte Bennassar Health Center, Palma de Mallorca, Illes Balears Spain; Instituto de Investigación de Palma (IDISPA), Palma de Mallorca, Illes Balears Spain; Oslo University Hospital, Oslo, Norway; Field Epidemiology and Laboratory Training Program Pakistan (FELTP), Islamabad, Pakistan; Haukeland University Hospital, Bergen, Norway; St. Olav Hospital, Trondheim, Norway; Moyamba District Hospital, Moyamba, Moyamba Sierra Leone; Haraldsplass Diaconal Hospital, Bergen, Norway; University of Bergen, Bergen, Norway; MRIGlobal, Rockville, MD USA; Sandia National Laboratories, Alburquerque, USA; Stavanger University Hospital, Stavanger, Norway; Hospital Son Espases, Palma de Mallorca, Spain

**Keywords:** Ebola virus disease (EVD), Sierra Leone, Clinical features of suspected Ebola patients, Diagnostic validation

## Abstract

**Background:**

The last ebola virus disease (EVD) outbreak has been the most important since 1976. EVD cases decreased drastically in Sierra Leone at the beginning of 2015. We aim to determine the clinical findings and evolution of patients admitted to an Ebola treatment center (ETC) during the epidemic’s late phase.

**Methods:**

We analyze retrospectively data of patients admitted to the Moyamba ETC (December 2014-March 2015). Patients were classified in EVD or non-EVD patients according to the results of Ebola virus real-time reverse transcription polymerase chain reaction (ZAIRE-RT-PCR).

**Results:**

Seventy-five patients were included, 41.3 % were positive for ZAIRE-RT-PCR. More women (68 % vs 28 %, *p* = 0.001) were EVD-positive. More EVD patients had previous contact with an Ebola patient (74.2 % vs 36.3 %, *p* < 0.001). At admission, EVD patients were more likely to have fatigue (96.7 %, *p* < 0.001), diarrhea (67.7 %, *p* = 0.002), and muscle pain (61.3 %, *p* = 0.009); but only objective fevers in 35.5 % of EVD patients. The most reliable criteria for diagnosis were: contact with an Ebola patient plus three WHO symptoms (LR + =3.7, 95 % CI = 1.9–7.3), and positive contact (LR + =2.3, 95 % CI = 1.15–4.20). Only 45.2 % of EVD patients developed fevers during stay, but 75 % developed gastrointestinal symptoms. Non-EVD patients had gastrointestinal problems (33 %), respiratory conditions (26.6 %), and others such as malaria, HIV or tuberculosis with a mortality rate of 11.4 %. vs 58 % in EVD group (*p* < 0.001).

**Conclusions:**

More non-EVD patients were admitted in the outbreak’s late phases. The low percentage of initial fever highlights the need to emphasize the epidemiological information. EVD patients presented new symptoms getting worse and requiring closer follow-up. Diagnoses of non-EVD patients were diverse with a remarkable mortality, presenting a challenge for the health system.

## Background

A new Ebola treatment center (ETC) opened in the district of Moyamba (Sierra Leone) in December 2014. Since mid-August 2014, the district of Moyamba had only one Holding Center to manage patients with Ebola virus disease (EVD), and this center admitted 244 patients. The new ETC opened following collaboration of the Department for International Development (DFID), the Norwegian government, *Solidarités International* (France), Doctors of the World (UK), and *Médicos del Mundo* (Spain). The main aim of this ETC was to treat and manage EVD cases in response to the World Health Organization (WHO) declaration of EVD as public health emergency of international concern on 8 August 2014, demanding the need for “a coordinated international response to stop and reverse the international spread of Ebola because the Ebola outbreak in West Africa constitutes an extraordinary event” [1].

The creation of ETC facilities was one of the most important factors [2] that caused a significant decrease in the number of EVD cases during February and March 2015 throughout the country, including in the district of Moyamba, with fewer cases of confirmed EVD among suspected patients. For instance, during the week of 29 March 2015, there were 25 confirmed cases with EVD in Sierra Leone (33 during the previous week) [3]. Finally, on 7 November 2015, WHO declared Sierra Leone to be free of Ebola.

We examined patients with suspected but unconfirmed EVD who were admitted to the ETC during the final stages of this epidemic. To increase public health knowledge, this paper also covers information about the EVD negative patients admitted in Moyamba ETC.

The specific purposes of the present study were to:

- Compare the clinical characteristics of confirmed cases (EVD patients) and non-confirmed cases (non-EVD patients) treated at the ETC in Moyamba.
- Assess the diagnostic validity of initial symptoms used in WHO case definition to diagnose EVD in a low-incidence situation.
- Describe the symptoms of the patients during the stay in the ETC.
- Know information about the presumptive diagnosis of non-EVD patients.

## Methods

This is a retrospective observational study. The study population consists of all patients admitted for treatment at the Moyamba ETC from December 2014 to March 2015. All patients were referred to the ETC after an epidemiological investigation carried out by government epidemiological research teams who were specifically trained to apply WHO standard criteria for the diagnosis of EVD (Table 1) [4, 5]. To identify potential probable cases of EVD that would need special care and isolation during their admission, all patients were re-examined in the triage zone of the ETC. Patients were admitted to a pavilion for those with suspected EVD or a pavilion for those with probable EVD depending on clinical findings, history of contact with a confirmed case of EVD, or attendance at a funeral of a case with confirmed EVD. On admission, standardized treatment was started according to protocols based on the guidelines WHO and *Médecins Sans Frontieres* (MSF), later modified from the “Hastings protocol” [6]. Real-time reverse transcription polymerase chain reaction (ZAIRE-RT-PCR) for EVD was performed for all admitted patients, and those with positive results were immediately transferred to a pavilion designated for EVD confirmed patients.

**Table 1:** WHO case definitions for Ebola virus disease during an epidemic

A **suspect case** is any person:	
• Having had contact with a clinical case **AND**	
• Presenting with acute fever (>38 °C)	
**OR**	
• Having had contact with a clinical case (suspect, probable or confirmed) **AND**	
• Presenting with 3 or more of the symptoms below:	
**OR**	
• Presenting with acute fever **AND**	
• Presenting with 3 or more of the concerning symptoms below:	
○ Headache ○ Abdominal pain ○ Generalized or articular pain ○ Difficulty in swallowing ○ Intense fatigue ○ Difficulty in breathing	○ Nausea or vomiting ○ Hiccups ○ Loss of appetite ○ Miscarriage ○ Diarrhea
**OR**	
• Any person with unexplained bleeding or miscarriage	
**OR**	
• Any unexplained death.	

Adapted from: Gove S, Wurie A, IMAI-IMCI Alliance. Clinical management of patients in the ebola treatment centres and other care centres in Sierra Leone. Adaptation of the WHO generic. Ed MOHS. December 2014

The patient clinical variables that we examined were selected based on previous publications [7, 8]. History of symptoms were demanded at admission, and were registered (presence of fever or feverish at home, time since the onset of symptoms, government epidemiological teams information). Initial symptoms were screened once more at admission, taking the temperature in this moment. Information about treatments or chronic diseases was asked, but information about antipyretic medication was not always recorded. Clinical information was recorded in MS Word® for the clinical records and MS Excel® for the standardized information. All data were collected as part of the daily routine patient care and recorded on a paper based clinical charts and kept securely on standardized forms. Data extracted for research purposes were anonymised and stored in a password-protected database. The Sierra Leone Ethics and Scientific Review Committee and the Western Norwegian Regional Committee for Medical and Health Research Ethics approved the study.

Diagnostic services were provided by the US Centers for Disease Control (CDC) laboratory using the Ebola virus (Zaire) nucleoprotein and the real-time reverse transcription polymerase chain reaction (ZAIRE RT-PCR) in Bo (Sierra Leone) before 12 January 2015, and on-site by the US DoD MEDaC Laboratory thereafter.

Descriptive analysis was performed by calculating frequencies with proportions for categorical variables and means with standard deviations or medians and quartiles (depending on the distribution of variable) for continuous variables. To test the association between each variable and outcome (diagnosis of EVD), chi square/Fisher’s exact test or Student’s *t*-test/Mann–Whitney U test was used. A *p*-value less than 0.05 was considered statistically significant. Sensitivity, specificity, positive and negative likelihood ratios (LR+ and LR-), and positive and negative predictive values (PPV and NPV) were calculated with 95 % confident intervals (CIs) for each clinical variable and WHO criterion. Microsoft Excel® and IBM® SPSS® Statistics ver. 20 were used for data analysis.

Given the absence of complementary explorations in an ETC, the presumptive diagnosis in non-EVD patients were made by consensus of the clinician team based on symptoms and previous history. Some of these diagnoses were confirmed by district hospital during the days after discharge (blood tests, response to treatment).

## Results

### Basic characteristics of EVD and non-EVD patients

**Fig. 1:**
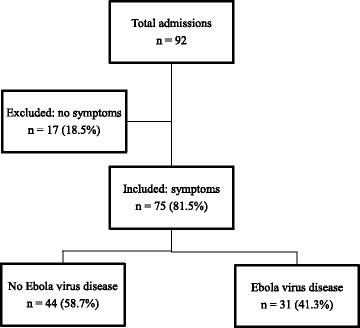
Disposition of patients admitted to the Ebola Treatment Center in Moyamba (Sierra Leone) from December 2014 to March 2015: Total admissions 92, excluded (no symptoms) 17 (18.5 %), included (symptoms) 75 (81.5 %), Non Ebola virus cases 44 (58.6 %) and Ebola virus cases 31 (41.4 %)

From December 2014 to March 2015, 92 patients were admitted to the ETC in Moyamba (Sierra Leone). A total of 81.5 % (75/92) were admitted because they had symptoms of EVD and 18.5 % (17/92) because they were relatives or caregivers of patients admitted with symptoms or had previous contact with an EVD patient. Individuals in this last group were RT-PCR negative for EVD in two test and were excluded from our analysis because they did not complain of any symptoms during their stays or during the 21 days period of isolation after their admission (Fig. 1). During this stage of the epidemic, there were more non-confirmed/suspected patients (non-EVD, *n* = 44, 58.7 %) than confirmed cases (EVD, *n* = 31, 41.3 %) (Fig. 1).

The overall mean patient age was 34 years (range: 0 months–80 years), and 66.7 % (50/75) were men. Table 2 shows characteristics of the non-EVD and EVD groups. More women were confirmed EVD patients 68 % (17/25) *vs*. 28 % (14/50) in men with significant differences (*p* = 0.001). Epidemiological information about contact history was unknown in 17 % (13/75) of the included patients. The EVD group had a higher percentage of individuals with a history of contact with confirmed patients (74.2 % *vs.* 36.3 %, *p* < 0.001), of individuals who met any WHO criterion for suspected EVD at admission (87 % *vs.* 59 %, *p* = 0.01), and of individuals who had one of three WHO criteria for suspected EVD at admission (contact and fever, contact and 3 symptoms, and unexplained bleeding, *p* < 0.05 for each). The EVD group also had a longer median stay at the ETC (7 days [IQR: 3–12 days] *vs.* 4 days [IQR: 2–6 days], *p* = 0.004), and a greater mortality rate (58 % *vs.* 11.4 %, *p* < 0.001).

**Table 2:** Sociodemographic characteristics of patients with non-confirmed Ebola virus disease (non-EVD) and confirmed EVD who were treated in the Ebola Treatment Center in Moyamba (Sierra Leone) from December 2014 to March 2015

Total admissions	92		
Excluded (no symptoms)	17		
	Non-EVD Number (%)	EVD Number (%)	P value
Included patients	44 (58.7)	31 (41.3)	
Sex			
Male	36 (82)	14 (45.1)	
Female	8 (18)	17 (54.8)	0.001
Age (years)			
Median (1–3 Q)	40 (23–48)	30 (17–40)	0.095
Range (months-years)	0–80	0–85	
History of contact with an Ebola patient			
Yes	16 (36.3)	23 (74.2)	<0.001
No	23 (52.3)	0 (−)	
Unknown	5 (11.3)	8(25.8)	
Suspect criteria			
Contact + Fever	10 (22.7)	15 (48.4)	0.026
Contact + 3 symptoms	8 (18)	21 (67.7)	<0.001
Fever + 3 symptoms	22 (50)	18 (58)	0.639
Bleeding	0 (−)	11 (35.5)	<0.001
Any WHO criterion	26 (59)	27 (87)	0.01
Onset symptoms (days)			
Median (1–3 Q)	2(1–6)	3 (1–5)	0.564
Stay length (days)			
Median (1–3 Q)	4 (2–6)	7(3–12)	0.004
State at discharge			
Alive	39 (88.6)	13 (42)	<0.001
Dead	5 (11.4)	18 (58)	

### Clinical features of EVD and non-EVD patients

The main initial symptoms of all suspected patients in Moyamba ETC were: fatigue 75 % (55/75), fever history or observed on admission 67 % (50/75), anorexia 48 % (36/50), diarrhea, abdominal pain and headache with 45 % (34/75) each, joint pain 44 % (33/75), muscle pain 43 % (32/75) and vomiting 41 % (31/75).

Table 3 shows the presence of initial symptoms and the development of symptoms during the stay at the ETC for patients in the EVD and non-EVD groups, with significant factors. This analysis indicates that patients in the EVD group were significantly more likely to have fatigue (96.7 % *vs.* 56.8 %, OR = 22.8, *p* < 0.001), diarrhea (67.7 % vs. 29.5 %, OR = 5, *p* = 0.002), muscle pain (61.3 % *vs.* 29.5 %, OR = 3.8, *p* = 0.009), vomiting (58 % vs. 29.5 %, OR = 3.3, *p* = 0.018), dysphagia (eight patients vs. none, OR = 0.3, *p* < 0,001) and bleeding (11 patients vs. none, OR = 0.3, *p* < 0.001). Fever was measured at admission and every day until the discharge, only 35.5 % of EVD patients and 34 % of non-EVD patients was observed having fever at admission. But if we join patients with objective fever and those with history of fever at home, the percentages increase to 61.3 % of EVD patients and 70.4 % of non-EVD patients.

**Table 3:** Initial symptoms and evolution of symptoms in patients with non-confirmed Ebola virus disease (non- EVD) and confirmed EVD who were treated at the Ebola Treatment Center in Moyamba (Sierra Leone) from December 2014 to March 2015

	Initial symptoms				Evolution symptoms			
Symptoms	Non-EVD *N* = 44 Number (%)	EVD *N* = 31 Number (%)	Unadjusted odds ratio (95 % CI)^a^	P value	Non-EVD *N* = 44 Number (%)	EVD *N* = 31 Number (%)	Unadjusted odds ratio (95 % CI)	P value
Fever^b^	31 (70.4)	19 (61.3)	0.6 (0.2–1.7)	0.461				
Objective Fever (>38 °C)	15(34)	11 (35.5)	1 (0.4–2.6)	0.585	11 (25)	14 (45.2)	2.3 (0.9–6.6)	0.085
Vomiting	13 (29.5)	18 (58)	3.3 (1.3–8.7)	0.018	14 (43.7)	23 (74.2)	5.2 (2.2–17.2)	<0.001
Diarrhea	13 (29.5)	21 (67.7)	5 (1.8–13.5)	0.002	18 (40.9)	24 (77.4)	5 (1.8–14)	0.002
Fatigue	25 (56.8)	30 (96.7)	22.8 (2.8–182.4)	<0.001	29 (65.9)	31 (100)	15.5 (2–125.1)	0.001
Anorexia	17 (38.6)	19 (61.3)	2.5 (0.9–6.4)	0.064	19 (43.1)	27 (87)	8.9 (2.6–29.7)	<0.001
Abdominal pain	20 (45.4)	14 (45)	1 (0.4–2.5)	1	25 (56.8)	22 (71)	1.9 (0.7–5)	0.236
Chest pain	9 (20.4)	7 (22.5)	1 (0.4–3.5)	1	12 (27.2)	18 (58)	3.7(1.4–9.8)	0.009
Muscle pain	13 (29.5)	19 (61.3)	3.8 (1.4–9.9)	0.009	13 (29.5)	22 (71)	6 (2.7–16)	<0.001
Joint pain	17 (38.6)	16 (51.6)	1.7 (0.7–4.3)	0.346	19 (43.1)	19 (61.3)	2 (0.8–5.3)	0.161
Headache	17 (38.6)	17 (54.8)	1.9 (0.8–4.9)	0.239	24 (54.5)	18 (58)	1.1 (0.5–3)	0.816
Back pain	3 (6.8)	2 (6.4)	0.9 (0.1–5.8)	1	7(15.9)	7 (212.6)	1.5 (0.5–5)	0.552
Pain behind eyes	0 (0)	0 (0)	* (*)	* (*)	0 (0)	0 (0)	* (*)	* (*)
Cough	0 (0)	0 (0)	* (*)	* (*)	17 (38.6)	12 (38.7)	1 (0.4–2.5)	1
Breathing problems	0 (0)	0 (0)	* (*)	* (*)	7(15.9)	7 (21.9)	1.5 (0.5–4.9)	0.552
Dysphagia	0 (0)	8 (25.8)	*(*)	<0.001	6 (13.6)	16 (51.6)	6.7 (2.2–20.5)	0.001
Sore throat	0 (0)	0 (0)	* (*)	* (*)	2 (4.5)	1 (3.2)	0.7 (0.06–8)	1
Jaundice	3 (6.8)	0 (0)	* (*)	* (*)	4 (9)	0 (0)	* (*)	* (*)
Red eyes	8 (18)	12 (38.7)	2.8 (1–8.1)	0.065	8 (18)	15 (48.3)	4.2 (1.5–12)	0.01
Skin rash	1 (2.3)	1 (3.2)	1.4 (0.08–24)	1	1 (2.3)	1 (3.2)	1.4 (0.1–23)	1
Hiccups	6 (13.6)	2 (6.4)	0.4 (0.08–2.3)	0.457	6 (13.6)	2 (6.4)	0.4 (0.08–2.3)	0.457
Coma / unconscious	0 (0)	0 (0)	* (*)	* (*)	1 (2.3)	9 (29)	17.6 (2–147.8)	0.001
Confused / disoriented	4 (9)	1 (3.2)	0.3 (0.03–3)	0.397	7(15.9)	14 (45)	4.3 (1.5–12.7)	0.009
Bleeding	0 (0)	11 (35.5)	* (*)	<0.001	0 (0)	16 (51.6)	* (*)	<0.001

^a^ 95 % CI: 95 % confidence interval

^b^ If the patient reported having a fever or was >38 °C upon admission

* = OR and/or 95 % CI not applicable

### Diagnostic value of initial symptoms

Table 4 shows the diagnostic value of all initial symptoms, including WHO criteria, with significant factors. Upon admission, the highest sensitivities for confirmed EVD were for history of contact with an EVD confirmed person (100 %, 95 % CI: 100–100 %), and fatigue (96.8 %, 95 % CI: 90.6–100 %). The sensitivity was somewhat greater when assessing symptoms together, with 96.8 % (95 % CI: 90.6–100 %) for fatigue and anorexia and 83.9 % (95 % CI: 70.9–96.8 %) for digestive symptoms. The PPVs for initial symptoms were all relatively low except for presence of bleeding and dysphagia (100 %, 95 % CI: 100–100 %)/. Although the presence of any WHO criterion had a relatively high sensitivity (87.1 %, 95 % CI: 75.3–98.9 %), it had a low specificity (40.9 %, 95 % CI 26.4–55.4 %) and a low PPV (50.9 %, 95 % CI: 37.5–64.4 %). Based on LRs, the best diagnostic criteria were: *(i)* confirmed contact with an Ebola patient plus 3 WHO symptoms (LR+ = 3.7, 95 % CI = 1.9–7.3; LR- = 0.4, 95 % CI = 0.2–0.7), *(ii)* have had contact (LR+ = 2.3, 95 % CI = 1.15–4.20; LR- = 0.65, 95 % CI = 0.44–0.95), and *(iii)* diarrhea (LR+ = 2.3, 95 % CI = 1.4–3.8; LR- = 0.5, 95 % CI = 0.3–0.8).

**Table 4:** Diagnostic value of initial symptoms in patients admitted to the Moyamba Ebola Treatment Center

	Sensitivity (95 % CI)	Specificity (95 % CI)	Likelihood ratio positive (95 % CI)	Likelihood ratio negative (95 % CI)	PPV (95 % CI)	NPV (95 % CI)
Contact	100 (*)	59 (43.5–74.4)	2.4 (1.7–3.6)	*	59 (43.5–74.4)	100 (*)
Fever (≥38 °C or referred)	61.3 (44.1–78.4)	29.5 (16.1–43)	0.9 (0.6–1.2)	1.3 (0.7–2.5)	38 (24.5–51.5)	52 (32.4–71.6)
Vomiting	58.1 (40.7–75.4)	70.5 (57–83.9)	2 (1.1–3.4)	0.6 (0.4–1.0)	58.1 (40.7–75.4)	70.5 (57–83.9)
Diarrhea	67.7 (51.3–84.2)	70.5 (57–83.9)	2.3 (1.4–3.8)	0.5 (0.3–0.8)	61.8 (45.4–78.1)	75.6 (62.5–88.8)
Fatigue	96.8 (90.6–100)	43.2 (28.5–57.8)	1.7 (1.3–2.2)	0.1 (0–0.5)	54.5 (41.4–67.7)	95 (85.4–100)
Anorexia	61.3 (44.1–78.4)	61.4 (47–75.8)	1.6 (1–2.5)	0.6 (0.4–1)	52.8 (36.5–69.1)	69.2 (54.7–83.7)
Abdominal pain	45.2 (27.6–62.7)	54.5 (39.8–69.3)	1 (0.6–1.6)	1 (0.7–1.6)	41.2 (24.6–57.7)	58.5 (43.5–73.6)
Chest pain	22.6 (7.9–37.3)	79.5 (67.6–91.5)	1.1 (0.5–2.6)	1.0 (0.8–1.2)	43.8 (19.4–68.1)	59.3 (46.8–61.9)
Muscle pain	61.3 (44.1–78.4)	70.5 (57–83.9)	2.1 (1.2–3.5)	0.5 (0.3–0.9)	59.4 (42.4–76.4)	72.1 (58.7–85.5)
Joint pain	51.6 (34–69.2)	61.4 (47–75.8)	1.3 (0.8–2.2)	0.8 (0.5–1.2)	48.5 (31.4–65.5)	64.3 (49.8–78.8)
Headache	54.8 (37.3–72.4)	61.4 (47–75.8)	1.4 (0.9–2.3)	0.7 (0.5–1.2)	50 (33.2–66.8)	65.9 (51.3–80.4)
Back pain	6.5 (0–15.1)	93.2 (85.7–100)	0.9 (0.2–5.3)	1.0 (0.9–1.1)	40.0 (−2.9–82.9)	58.6 (47.0–70.1)
Dysphagia	25.8 (10.4–41.2)	100 (*)	*	0.7 (0.6–0.9)	100 (*)	65.7 (54.3–77)
Red eyes	38.7 (21.6–55.9)	81.8 (70.4–93.2)	2.1 (1–4.6)	0.7 (0.5–1)	60 (38.5–81.5)	65.5 (47.6–70.2)
Skin rash	3.2 (0–9.4)	97.7 (93.3–100)	1.4 (0.1–21.8)	1.0 (0.9–1.1)	50.0 (0–100)	58.9 (47.6–70.2)
Hiccups	6.5 (0–15.1)	86.4 (76.2–96.5)	0.5 (0.1–2.2)	1.1 (1.0–1.3)	25 (0–55)	56.7 (44.9–68.6)
Confused / disoriented	3.2 (0–9.4)	90.9 (82.4–99.4)	0.3 (0–3)	1.1 (1.0–1.2)	20.0 (0–55.1)	57.1 (45.5–68.7)
Bleeding	35.5 (18.6–52.3)	100 (*)	*	0.6 (0.5–0.8)	100.0 (*)	68.8 (57.4–80.1)
Fatigue/anorexia	96.8 (90.6–100)	38.6 (24.2–53.0)	1.10 (0.79–1.55)	0.83 (0.44–1.57)	43.8 (29.7–57.8)	63.0 (44.7–81.2)
Digestive symptoms	83.9 (70.9–96.8)	45.5 (30.7–60.2)	1.54 (1.13–2.10)	0.35 (0.15–0.84)	52.0 (38.2–65.8)	80.0 (64.3–95.7)
Musculoskeletal pain	67.7 (51.3–84.2)	38.6 (24.2–53.0)	1.10 (0.79–1.55)	0.83 (0.44–1.57)	43.8 (29.7–57.8)	63.0 (44.7–81.2)
Headache / Pain behind eyes	54.8 (37.3–72.4)	61.4 (47.0–75.8)	1.42 (0.87–2.32)	0.74 (0.47–1.16)	50.0 (33.2–66.8)	65.9 (51.3–80.4)
Neurological symptoms	3.2 (0–9.4)	90.9 (82.4–99.4)	0.35 (0.04–3.02)	1.06 (0.95–1.19)	20.0 (0–55.1)	57.1 (45.5–68.7)
Contact + Fever	48.4 (30.8–66)	77.3 (64.9–89.7)	2.1 (1.1–4.1)	0.7 (0.5–1)	60 (40.8–79.2)	68.0 (55.1–80.9)
Contact + 3 symptoms	67.7 (51.3–84.2)	81.8 (70.4–93.2)	3.7 (1.9–7.3)	0.4 (0.2–0.7)	72.4 (56.1–88.7)	78.3 (66.3–90.2)
Fever + 3 symptoms	58.1 (40.7–75.4)	50 (35.2–64.8)	1.2 (0.8–1.8)	0.8 (0.5–1.4)	45 (29.6–60.4)	62.9 (46.8–78.9)
Any WHO criterion	87.1 (75.3–98.9)	40.9 (26.4–55.4)	1.5 (1.1–1.9)	0.3 (0.1–0.8)	50.9 (37.5–64.4)	81.8 (65.7–97.9)

*NPV* negative predictive value, *PPV* positive predictive value

* = 95%CI not applicable

### Evolution of patients

During the stay in the ETC, patients in the EVD group were significantly more likely than the non-EVD group to have symptoms of dysphagia (51.6 % *vs.* 13.6 %, OR = 6.7, *p* = 0.001), vomiting (74.2 % *vs.* 43.7 %, OR = 5.2, *p* = 0.001), muscle pain (71 % vs. 29.5 %, OR = 6 *p* < 0.001), or bleeding (51.6 % vs zero, *p* < 0.001), and additional symptoms of chest pain (58 % *vs.* 27.2 %, OR = 3.7, *p* = 0.009), red eyes (48.3 % *vs.* 18 %, OR = 4.2, *p* = 0.01), coma/unconsciousness (29 % *vs.* 2.3 %, OR = 17.6, *p* = 0.001), and confusion/disorientation (45 % *vs.* 15.9 %, OR = 4.3, *p* = 0.01). EVD patients developed fever only in 45.2 % (14/31) without differences with non-EVD group. In the subgroup of patients without initial fever (objective or referred) EVD patients developed fever in 50 % (6/12) without differences with non-EVD group, 46.2 % (6/13).

A presumptive diagnosis was possible in 68 % (30/44) of the non-EVD patients. Gastrointestinal and hepatic conditions were the most frequent presumptive diagnostic conditions (33.3 %, 10/44), followed by respiratory problems (26.6 %, 8/44), malaria (13.3 %, 4/44), previously known HIV (8.8 %, 3/44), and clinical Pott’s disease, diabetes mellitus, urethral syndrome, abdominal tumor, or cardiac conditions (2.3 % and 1 case each). More than two-thirds of all admitted patients (69.7 %, 53/76), recovered and were discharged. Non-EVD patients were more likely to recover and be discharged (88.6 % *vs.* 42 %, *p* < 0.001). Most non-EVD patients (68 %, 30/44) were sent home or to a convalescent or quarantine center, 9 were transferred to the Moyamba District Hospital, and 5 died at the ETC. All patients treated at the Moyamba District Hospital were discharged to their homes and were in good health several days after transfer.

## Discussion

International help for the 2014 Ebola epidemic arrived late in Moyamba, as in other places in Sierra Leone, and opening a new ETC in the field was difficult. Between August and December 2014, 244 patients were isolated at the small Holding Center in Moyamba, and 149 of them had confirmed EVD. Between December 2014 and March 2015, 75 patients were admitted for treatment to the ETC in Moyamba, and 42 % of them had confirmed EVD. At this ETC, as in others constructed in Sierra Leone, the number of patients admitted never reached the center’s capacity (100 beds). The Sierra Leonean Minister of Health declared the district free of Ebola in April 2015, and we have detailed clinical information of all patients treated at this ETC. Unlike other ETCs, the ETC in Moyamba treated all EVD-positive patients and suspected patients or contacts due to directives from the District Ebola Response Center (DERC) [9]. The striking decrease in incidence of EVD beginning in February 2014 led to changes in the ETCs, because many suspected cases required isolation until their statuses were confirmed. At that time, we decided to assess the diagnostic validity of EVD symptoms in a low incidence situation and to follow the presumptive diagnoses, evolution, and discharge of non-EVD patients.

Our study shows that in Moyamba ETC females were more likely to have EVD than males (17/25, 68 % *vs.* 14/50, 28 %, *p* = 0.001). This study also highlights the importance of determining the detailed epidemiological histories of individuals admitted to an ETC and whether they were exposed to patients with EVD between 2 and 21 days before presentation. In patients where the epidemiological information was known, our data showed that 74.2 % of EVD patients (but only 36 % of non-EVD patients) had previous contact with an affected patient. Other studies have also emphasized the importance of previous contact with EVD patients [10]. Lado et al. reported much lower percentages of EVD-positive subjects following contact with infected individuals, (but with less detailed observations and follow-up) [11].

The presence of fatigue, digestive symptoms (diarrhea, vomiting, dysphagia), muscle pain, and bleeding were the most common initial symptoms of EVD patients in our center, as in previous studies [5, 10, 12, 13]. However, in our study a relatively low percentage of EVD patients had objective fever at admission and during their stays at the ETC, without statistical differences between the two groups of patients. This seems surprising as fever was one of the major criteria for diagnosis of EVD, and is even considered a very important part of WHO criteria for suspicion of EVD. Schieffelin [4] and other researchers observed even lower percentages of objective fever in Ebola patients. These low rates of fever may partly be explained by the widespread administration of paracetamol to admitted patients.

The algorithms developed to identify suspected cases of EVD were based on definitions as established by the WHO and a consensus of experts [14]. However, the reliability of this approach for diagnosis of EVD has not been established [14]. In our study, the WHO criteria had acceptable sensitivity (87.1 %), but low specificity (40.9 %) and low PPV (50.9 %), as in other studies [10]. The most reliable criteria were contact with an infected person plus three symptoms (LR+ of 3.7 and NPV of 78.3 %), diarrhea (LR+ of 2.3 and NPV of 75.6 %), and contact with an infected person and fever (LR+ of 2.1 and NPV of 68 %). As in other studies [9, 10], unexplained bleeding at presentation had high specificity (100 %), but low sensitivity (35.5 %). Other authors have attempted to improve the suspected case definition using diagnostic scores, but achieved no improvement in specificity [10]. Taken together, these results emphasize the limitations of these diagnostic criteria and the need for fast and reliable laboratory tests upon admission to an ETC [15–17].

One of the important aspects of our study is that we followed the evolution of EVD and non-EVD patients following admission to the ETC. As expected, some symptoms became more common while patients were in the ETC. About 75 % of the EVD patients had increased gastrointestinal symptoms (vomiting, diarrhea, dysphagia or anorexia) while in the ETC, considered by Hunt et al. [9] to be a sign of stage-2 disease. Pain in different locations (muscular, chest pain) also became more common among admitted patients. Moreover, 50 % of admitted patients had symptoms that implied more severe stage-3 disease [9], namely bleeding/hemorrhage and neurological symptoms (confusion or coma). Previous studies reported that less than 15 % of patients with EVD presented with bleeding [4, 10, 11, 16], but 35.5 % of EVD patients in ETC Moyamba had bleeding on admission and 51.6 % developed bleeding during their stays at the ETC. This was related to a poor prognosis [13].

Patients admitted to the ETC who did not have EVD had diverse clinical conditions, with a predominance of gastrointestinal problems, respiratory problems, and malaria. Some of the non-EVD patients had clinical Pott’s disease, known HIV infection, or possibly cardiac conditions (clinically compatible with myocardial infarction). Up to 11.4 % of these patients died at the ETC (a remarkable part of overall mortality), one patient with known HIV infection, another patient with respiratory problem, a man with cardiac condition and two patients for unknown reasons. These findings point to the complexities involved in handling non-EVD patients at an ETC during an epidemic.

In our series, the death rate of patients with EVD was 58 %. In other studies of EVD the death rates were between 37 and 74 % [4, 9, 11, 18], although some studies reported that more than 50 % of patients were lost to follow-up [14]. Recent articles highlighted the uncertainty as to whether the clinical management of patients in this epidemic could have been improved, and what should be done in future epidemics [19, 20]. The training of healthcare staff in ETCs and the treatment protocols used in ETC Moyamba and in most ETCs were based on guidelines developed by the WHO and *Médecins Sans Frontieres*. These guidelines emphasize use of quarantine and epidemic control, but patient care is based on patient history and examinations. The examination, however may be difficult to perform and will be incomplete. Other studies [9, 19–23] have shown that more than 30 % of the EVD patients had acute renal insufficiency and abnormal potassium concentrations, and the association of acute renal insufficiency, hepatitis, and rhabdomyolysis with death from EVD demonstrates the need to monitor electrolytes and other laboratory parameters to improve the management and care of such patients.

### Limitations of the study

The main limitation of this study is its retrospective design. The work was done in a complicated clinical scene, where it could be difficult to obtain certain information. Data analysis was done on charts that were filled by different healthcare workers, and some information could be lacking. We know that the small number of patients and the participation of only one ETC in this study, limit the capability to generalize our conclusions to other ETCs, but we think our data give some useful information to the actual knowledge of the disease and we show the difficulties in performing complete clinical diagnoses at the ETC and the district hospital to which non-EVD patients were transferred.

## Conclusion

Our study shows a low specificity and PPV for WHO criteria in the late phases of the epidemics with more non-EVD patients admitted in an ETC. The low percentage of patients with fever at admission and during their stay at the ETC, our study highlights the importance of collecting detailed and complete epidemiological histories of patients with suspected EVD.

Our study detects a development of new symptoms and increase of initial symptoms related with a poor prognosis of most EVD patients after admission to the ETC. Therefore, such patients must be followed as closely as possible, although this can be difficult in the presence of an Ebola epidemic. Our presumptive diagnoses of non-EVD patients were diverse indicating that the ETCs and district hospitals must be better prepared and interrelated for a proper care of all suspected patients during the final phases of an Ebola epidemic.

We believe it may be advisable to modify some of the protocols used to care for patients admitted to an ETC. In particular, monitoring of electrolytes and other laboratory parameters may allow better management of patients with EVD and provide a safer and more appropriate method of care.

To improve the relationship between ETCs and health facilities (district hospitals, health centers), in the late phases of an outbreak, is a big challenge to assure a correct care to the Non-EVD patients.

## Abbreviations

CDC, Centers for Disease Control; CI, confidence interval; Ct, cycle threshold; DERC, District Ebola Response Center; DFID, Department for International Development; DoD, Department of Defense; ETC, Ebola Treatment Center; EVD, ebola virus disease; FDA, Food and Drug Administration; HIV, Human Immunodeficiency Virus; MSF, *Médecins Sans Frontieres*; NPV, negative predictive value; PCR, polymerase chain reaction; PPV, positive predictive value; WHO, World Health Organization
